# Structural and molecular characteristics of weight‐bearing volar skin can be reconstituted by micro skin tissue column grafting

**DOI:** 10.1096/fj.202400866R

**Published:** 2024-08-06

**Authors:** Christiane Fuchs, Ying Wang, Emma Wise, William A. Farinelli, R. Rox Anderson, Sunghun Cho, Jon H. Meyerle, Joshua Tam

**Affiliations:** ^1^ Wellman Center for Photomedicine Massachusetts General Hospital Boston Massachusetts USA; ^2^ Department of Dermatology Harvard Medical School Boston Massachusetts USA; ^3^ Department of Dermatology Uniformed Services University of the Health Sciences Bethesda Maryland USA; ^4^ Department of Dermatology Walter Reed National Military Medical Center Bethesda Maryland USA; ^5^ Harvard‐MIT Health Sciences and Technology Massachusetts Institute of Technology Cambridge Massachusetts USA

## Abstract

For patients with lower limb amputations, prostheses are immensely helpful for mobility and the ability to perform job‐related or recreational activities. However, the skin covering the amputation stump is typically transposed from adjacent areas of the leg and lacks the weight‐bearing capacity that is only found in the specialized skin covering the palms and soles (a.k.a. volar skin). As a result, the skin tissue in direct contact with the prosthesis frequently breaks down, leading to the development of painful sores and other complications that limit, and often preclude, the use of prostheses. Transplanting volar skin onto amputation stumps could be a solution to these problems, but traditional skin transplantation techniques cause substantial morbidity at the donor site, such as pain and scarring, which are especially problematic for volar skin given the critical functional importance of the volar skin areas. We previously developed the technology to collect and engraft full‐thickness skin tissue while avoiding long‐term donor site morbidity, by harvesting the skin in the form of small (~0.5 mm diameter) cores that we termed “micro skin tissue columns” (MSTCs), so that each donor wound is small enough to heal quickly and without clinically appreciable scarring or other long‐term abnormalities. The goal of this study was to establish whether a similar approach could be used to confer the structural and molecular characteristics of volar skin ectopically to other skin areas. In a human‐to‐mouse xenograft model, we show the long‐term persistence of various human plantar MSTC‐derived cell types in the murine recipient. Then in an autologous porcine model, we harvested MSTCs from the bottom of the foot and transplanted them onto excision wounds on the animals' trunks. The healing processes at both the donor and graft sites were monitored over 8 weeks, and tissue samples were taken to verify volar‐specific characteristics by histology and immunohistochemistry. The volar donor sites were well‐tolerated, healed rapidly, and showed no signs of scarring or any other long‐term defects. The graft sites were able to maintain volar‐specific histologic features and expression of characteristics protein markers, up to the 8‐week duration of this study. These results suggest that MSTC grafting could be a practical approach to obtain autologous donor volar skin tissue, confer volar skin characteristics ectopically to nonvolar skin areas, improve the load‐bearing capacity of amputation stump skin, and ultimately enhance mobility and quality‐of‐life for lower limb amputees.

## Graphical abstract

Using autologous, volar‐derived micro skin tissue columns (MSTCs) to convey volar skin characteristics ectopically to nonvolar skin sites. (A) In the human‐to‐mouse xenograft model, MSTCs from human plantar skin are implanted into full‐thickness wounds on immunodeficient mice. (B) In the porcine model, autologous MSTCs are harvested from the weight‐bearing plantar surface of each donor animal (volar MSTCs shown in photograph with ruler), then applied to full‐thickness excision wounds on the trunk skin overlying the thoracic cage in the same animal.

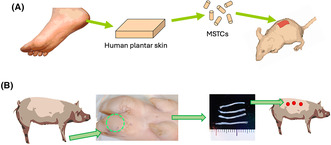

**Abbreviations**
DEJ: Dermal‐epidermal junction
H&E: Hematoxylin and eosin
IHC: Immunohistochemistry
MGH: Massachusetts General Hospital
MSTC(s): Micro skin tissue column(s)
STIM1: Stromal interaction molecule 1
STSG: Split‐thickness skin graft

## 1 INTRODUCTION

The use of prostheses after lower extremity amputation is often limited or even precluded by skin breakdown, abrasions, and other dermatoses that tend to develop at the stump‐prosthetic interface, which affect up to 74% of patients using lower‐limb prostheses.^1^ The most common skin problems associated with the use of prostheses are allergic reactions, bacterial or fungal infections, pressure ulcers, epidermoid cysts, calluses, and verrucous hyperplasia.^2^ Many of these problems are thought to arise because stump skin, unlike the skin covering the palms and soles (also known as volar skin), was never evolved for weight bearing. Volar skin is highly specialized in architecture and molecular composition,^3^, ^4^, ^5^, ^6^ and these unusual properties of volar skin are thought to convey unique advantages for weight bearing,^7^ such that volar skin is able to tolerate pressures and sheer stresses about 200x higher than loads that would cause the breakdown of skin covering other parts of the body.^8^ Classic heterotypic transplantation and in vitro co‐culture experiments have shown that key structural characteristics in the volar epidermis can be maintained as long as the underlying dermis is included in a skin graft,^9^ and that the unique expression of keratin 9 in the volar epidermis is induced and maintained by paracrine signaling from fibroblasts in the volar dermis.^10^, ^11^ These results also indicate that most volar skin characteristics are innate to volar skin, as opposed to merely a generic response induced by mechanical loading of the skin. This has led to interest in the novel concept of “volarizing” stump skin—that is, conferring volar skin characteristics ectopically onto the amputation stump—in order to increase the latter's ability to withstand mechanical stress.^8^, ^12^, ^13^ However, autologous transplantation of volar skin is impractical as a clinical solution, since the morbidities caused by traditional skin harvesting techniques at the donor site, including long‐lasting pain and scarring, are much less tolerable in the highly sensitive and functionally important volar skin areas. Thus current efforts at conferring volar characteristics ectopically have focused on harvesting and expanding fibroblasts from the volar dermis, then implanting them into nonvolar skin sites, with the goal of inducing volar characteristics in the overlying epidermis.^8^, ^12^, ^13^ This approach is the subject of an ongoing clinical study.^14^

**FIGURE 1:**
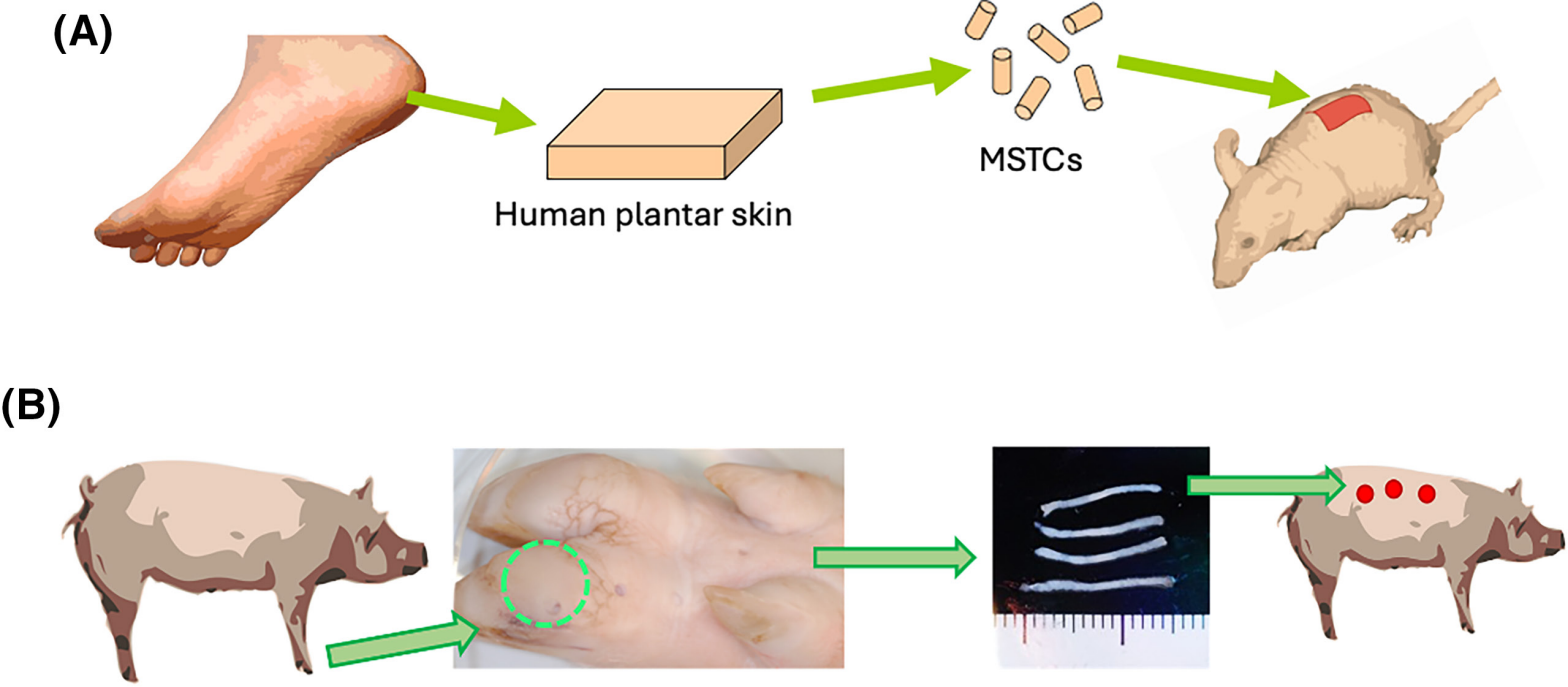
Study outline. (A) In the human‐to‐mouse xenograft model, MSTCs from human plantar skin are implanted into full‐thickness wounds on immunodeficient mice. (B) In the porcine model, autologous MSTCs are harvested from the weight‐bearing plantar surface of each donor animal (volar MSTCs shown in photograph with ruler), then applied to full‐thickness excision wounds on the trunk skin overlying the thoracic cage in the same animal.

We previously developed a method for harvesting and applying full‐thickness skin in the form of micro skin tissue columns (MSTCs), with the key feature being each MSTC is small enough (<1 mm diameter) that the donor sites could heal spontaneously without long‐term pain or scarring,^15^ thereby circumventing the most problematic drawback of autologous skin grafting. This minimization of donor site morbidity has been independently confirmed in clinical testing, where subjects reported pain scores averaging 1 or less (on a 10‐point scale) only for the first 2 days after harvesting, with no pain afterward and no donor site scarring.^16^ MSTCs could be directly applied into wound beds (in a manner similar to minced skin grafting) to replenish all major cutaneous cell populations and accelerate wound healing.^17^, ^18^ Given that full‐thickness skin transplants (defined as including both the epidermis and the entire depth of the dermis) are known to be able to sustain characteristics of their original donor sites,^9^ we reasoned that full‐thickness MSTCs may similarly be able to stably confer volar characteristics to other skin areas, while avoiding the donor site morbidities associated with traditional skin grafting techniques. The objective of the current study is to establish whether the structural and molecular characteristics of volar skin could be recapitulated ectopically by harvesting MSTCs from volar skin and transplanting them into nonvolar skin wounds, without causing long‐term donor site morbidities (Figure 1).

## 2 MATERIALS AND METHODS

### 2.1 Animal procedures

All animal procedures were performed in accordance with the Public Health Service Policy on Humane Care and Use of Laboratory Animals and with approval from the MGH Institutional Animal Care and Use Committee.

#### 2.1.1 Human‐to‐mouse xenograft model

The human‐to‐mouse xenograft model was performed as previously described.^17^ Briefly, a fully deidentified human plantar skin tissue sample that would otherwise be discarded was obtained from the Massachusetts General Hospital (MGH) Pathology Department, with approval by the MGH Institutional Review Board. The skin tissue was disinfected by soaking in a solution of 100 U/mL penicillin–streptomycin and 2.5 μg/mL Amphotericin B in normal saline, for 5 min. MSTCs of full‐thickness plantar skin tissue were collected using a custom‐built harvester with a 19G harvesting needle (corresponding to an inner diameter of approximately 700 μm), as described in detail elsewhere.^19^ The human plantar MSTCs were applied directly into full‐thickness skin wounds of approximately 1 × 1cm^2^ that were produced by excision on the dorsal skin of two adult female hairless mice with severe combined immunodeficiency (Crl:SHO‐*Prkdc* ^*scid*^ *Hr* ^*hr*^, Charles River Laboratories). The wounds were then protected by spraying on a layer of liquid barrier film (Cavilon, 3M), followed by Tegaderm film dressing (3M), and wrapping with an elastic bandage. This dressing regimen was maintained until an epidermal barrier became visible, and the animals were euthanized after 8 weeks for tissue collection.

#### 2.1.2 Autologous porcine model

Two adult female Yorkshire swine, each about 35 kg at the time of acquisition, were used. Full‐thickness MSTCs, each approximately 700 μm in diameter, were harvested from the animals' volar skin using custom‐made 19G harvesting needles.^19^ During harvesting, we took care to avoid overlapping donor sites (since donor site size is the critical determinant of whether donor site scarring occurs after MSTC harvesting), while maximizing the amount of donor tissue obtained. In order to minimize potential negative impacts on the animals' mobility, the harvesting procedure was only done on one foot in each animal. After completion of harvesting, a surgical skin adhesive was applied over the donor wounds (Dermabond Advanced, Ethicon, Raritan, NJ), then the foot was bandaged. Separately, 1.5 cm diameter full‐thickness wounds were produced on the animal's trunk by excising down to the subcutaneous fat. Wound margins were marked with tattoos. The volar MSTCs were applied “randomly” (i.e., without attempting to maintain the epidermal‐dermal orientation) into the wound bed. The exact number of MSTCs was not counted, but they were applied at roughly 20% of the excised tissue mass. Control wounds of similar size and depth were allowed to heal by secondary intention. Control and treatment wounds were alternated spatially to compensate for anatomical variations in skin properties. Wounds were dressed with a combination of hydrogel and foam dressings (Tegaderm, 3 M Health Care, St. Paul, MN), followed by an additional adhesive dressing (DuoDERM CGF, ConvaTec, Greensboro, NC), a stockinette, and a nylon jacket. In total, eight volar MSTC‐treated and seven control wounds were distributed evenly across two different animals. Wounds and donor sites were inspected and photographed weekly for 4 weeks, then biweekly until end of study at 8 weeks post‐wounding, when tissue biopsies were taken from both donor and graft sites for additional study. Wound contraction was evaluated from the photographs (Figure S3).

### 2.2 Histology

Skin biopsies were fixed in 4% formaldehyde and then embedded in paraffin. Five micrometer sections were stained with hematoxylin and eosin (H&E) and Gömöri's Trichrome for general histology. Immunohistochemical staining was performed as previously described,^20^ using antibodies against Keratin 7 (Abcam, ab68460, 1:100), Keratin 9 (Abcam, ab171966, 1:200), Vimentin (Vector Laboratories, VP‐RM17, 1:100), S100A8 (MyBiosource, MBS2028565, San Diego, CA, 1:400), S100A12 (Novus Biologicals, NBP1‐86694, Centennial, CO, 1:250), S100A14 (ThermoFisher, PA5‐55666, Waltham, MA, 1:2500), and STIM1 (Novus Biologicals, NBP110‐60547, Centennial, CO, 1:200). Stained slides were scanned into a digital format using the Nanozoomer (Hamamatsu, Bridgewater, NJ). Quantitative analysis was performed using Fiji,^21^ including the Color Deconvolution 2 plugin.^22^, ^23^ Additional details on the interpretation and analysis of histological data are included in the supplement (Figures S1, S2, S4 and S5).

### 2.3 Statistical analysis

The quantified histologic results were compared between MSTC‐treated and control groups by the Student's t‐test, using GraphPad Prism Version 9.5.1 for macOS (GraphPad Software, San Diego, CA, www.graphpad.com). A value of *p* ≤ .05 was considered statistically significant.

## 3 RESULTS

### 3.1 Persistence of MSTC‐derived human plantar skin cells in murine recipients

**FIGURE 2:**
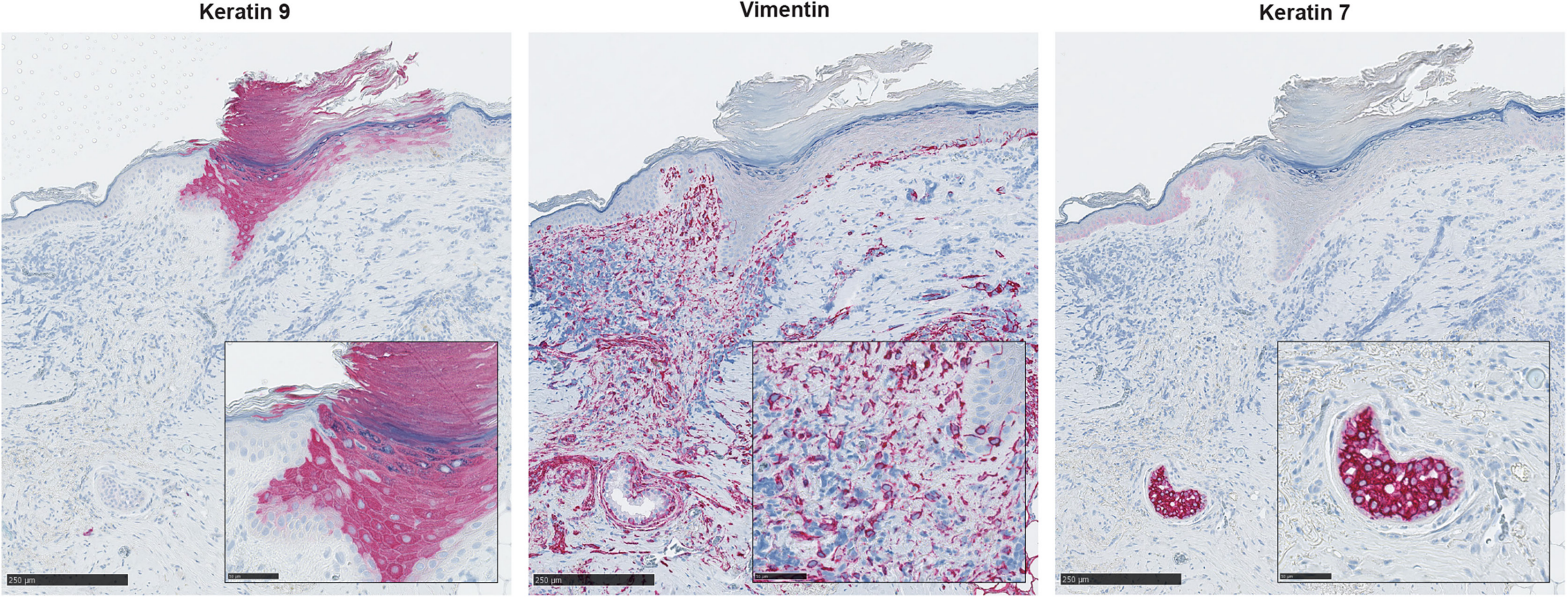
Persistence of human plantar MSTC‐derived cells in murine recipients. Eight weeks after MSTCs were applied to full‐thickness excision wounds in immunodeficient mice, human cells were labeled by immunohistochemistry using human‐specific antibodies (positive staining shown in red). These include human plantar keratinocytes expressing the volar marker keratin 9, human fibroblasts expressing vimentin, and eccrine sweat glands expressing keratin 7, as labeled in the figure. Images shown are from consecutive sections. All three antibodies are human‐specific and do not cross‐react with murine tissue (Figure S2). Insets show high‐power views of the targets of interest. Scale bars: 250 μm, insets: 50 μm.

While the MSTC approach is ultimately intended for autologous skin grafting, for experimental purposes it is difficult to discern MSTC‐derived cells in the autologous setting. Therefore, to establish the ability of volar MSTC‐derived cells to persist in vivo, we harvested MSTCs from post‐surgical human plantar skin tissue that would otherwise be discarded and applied them into full‐thickness wounds on mice (Figure 1A). The tissue was harvested 8 weeks later and stained with human‐specific antibodies to confirm the presence of human keratinocytes expressing the volar‐specific marker keratin 9 in the epidermis, as well as keratin 7‐expressing eccrine sweat glands and vimentin‐positive human fibroblasts in the dermis (Figure 2). All the positive‐staining cells were of human origin, as the antibodies did not react with normal mouse tissue (Figure S2). These results confirm that MSTC‐derived volar skin cells are able to survive the transplantation process, integrate with recipient site tissue, and persist in vivo.

### 3.2 Differential structural and molecular characteristics between porcine volar and nonvolar skin

**FIGURE 3:**
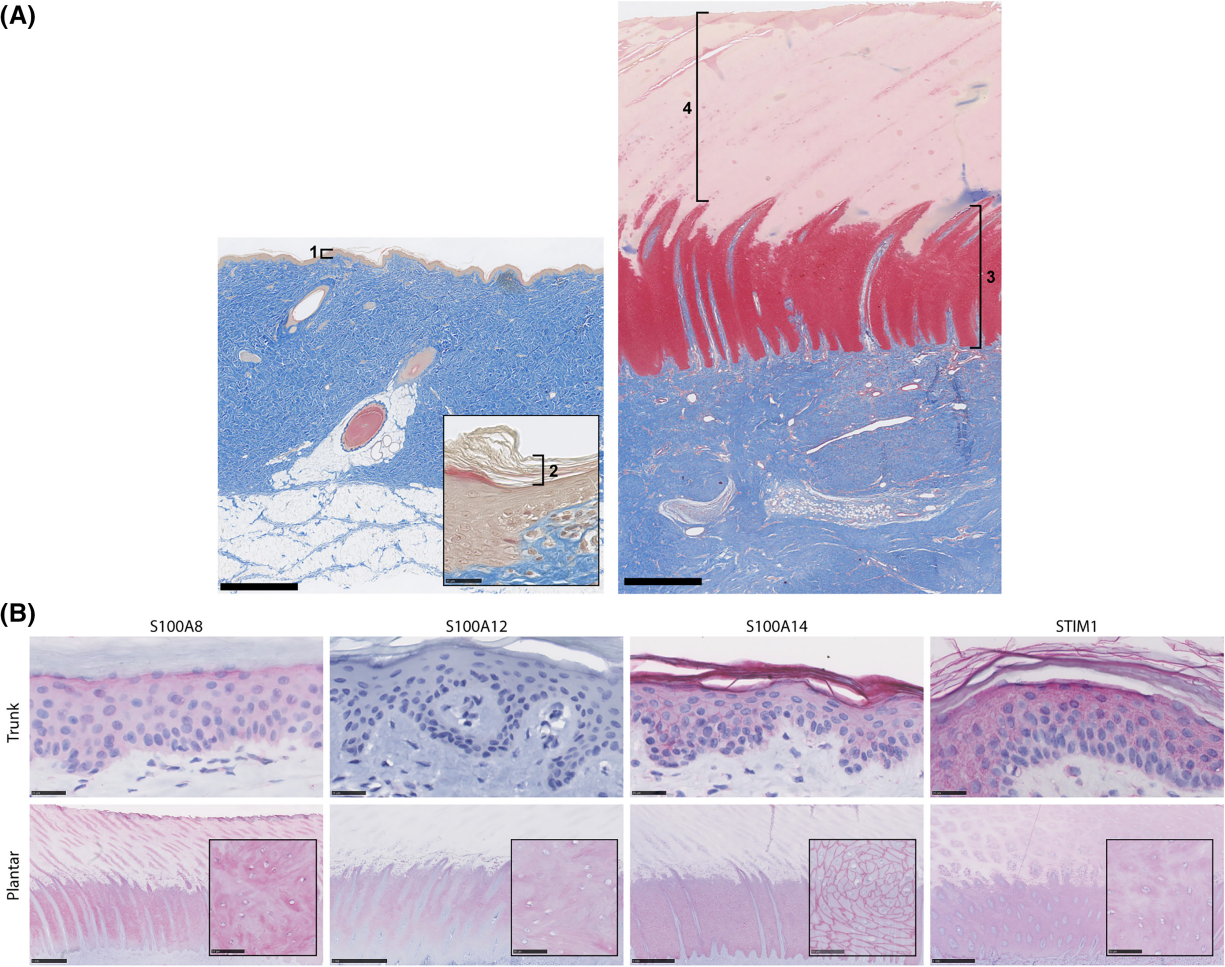
Distinctive structural and molecular characteristics of porcine volar skin. (A) Trichrome‐stained tissue sections from porcine trunk (left) and volar skin (right) are shown at the same magnification for comparison. Note the great difference in thickness between the epidermis in trunk skin (bracket #1) versus volar skin (brackets #3 and 4). The trunk stratum corneum is too thin to see in the main image and is therefore shown in the inset (bracket #2), displaying the classic layered structure. In contrast, the volar stratum corneum (bracket #4) is greatly thickened and has a compact structure. (B) IHC staining (red) showing differential expression of marker proteins in trunk versus volar (plantar) skin, as labeled in the figure. Porcine volar skin is distinguished by upregulation of S100A8, expression of S100A12, altered spatial distribution of S100A14 (strong pericellular staining in the viable epidermis with weak staining in the stratum corneum in plantar skin, vs. strong stratum corneum staining in trunk skin) and downregulation of STIM1. Insets show the plantar epidermis (specifically the *stratum spinosum*) at higher magnification. Scale bars: A: 1 mm; inset, 25 μm; B: Trunk, 25 μm; plantar, 1 mm; insets, 50 μm.

For more detailed evaluations of wound treatment and donor site healing outcomes, we chose the porcine model due to the broad similarities in anatomy, biochemistry, and the wound healing process between human and pig skin.^24^, ^25^, ^26^ We have previously found that, as is the case in human volar skin, porcine volar skin is characterized by unique structural and molecular characteristics.^20^ The volar epidermis is characterized by substantially increased thickness, hypertrophic keratinocytes, prominent rete ridges (the interdigitations at the dermal‐epidermal junction that anchor the two layers of skin to each other), and a *stratum corneum* (the uppermost, devitalized layer of the epidermis) composed of corneocytes that have retained their cell volumes and are tightly interconnected in a mosaic pattern, as opposed to the classic (nonvolar) architecture of orderly, loosely connected layers of squamified (flattened) corneocytes (Figure 3A). Volar skin also has a unique molecular profile, that in pigs includes upregulation of many members of the S100 family, and reduced STIM1 protein (Figure 3B). Many of these features are consistent with characteristics previously described for human volar skin,^3^, ^4^, ^5^ with the notable exception of pigs lacking keratin 9, which is the volar‐specific skin marker in humans (and many other species).^27^ In this study, we harvested MSTCs from the animals' volar (plantar) skin and then transplanted the autologous MSTCs into excision wounds on the animals' trunks (Figure 1B). Healing of the donor and recipient sites was monitored for 8 weeks, then tissue samples were collected for further analyses. The aforementioned distinctive structural and molecular composition of porcine volar skin are used as benchmarks to verify that volar characteristics are conferred to ectopic skin areas. Post‐harvest donor site healing is an additional area of focus, as donor site morbidity is of particular concern for volar skin, due to the relatively small area and high functional importance of the volar skin regions.

### 3.3 Volar donor sites were well‐tolerated and healed without clinical signs of scarring

**FIGURE 4:**
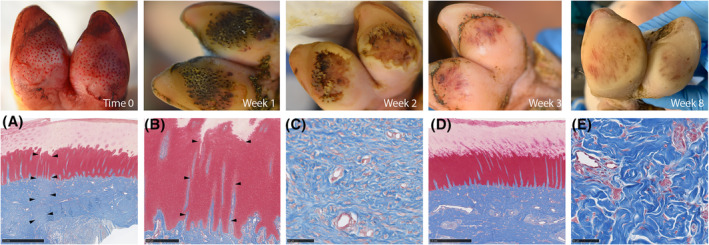
Donor site healing. Top row: Photographs of volar MSTC donor sites taken at various timepoints post‐harvest. Erosion of the stratum corneum was observed at week 2, which spontaneously resolved by week 3. Bottom row: Donor site histology by trichrome staining. (A) Full‐thickness view of a plantar donor site taken 8 weeks after initial MSTC harvesting. One MSTC donor site is outlined by arrow heads. (B) High‐power view of the same MSTC donor site (outlined by arrow heads). Rete ridges within the donor site are similar in depth but thinner than rete ridges in the surrounding uninjured skin. (C) Dermal collagen in the MSTC donor site appears to be in the form of thinner fibrils. (D) Histology of uninjured porcine plantar skin shown at the same magnification as (A) for comparison. (E) Histology of dermal collagen in uninjured porcine plantar skin shown at the same magnification as (C). Scale bars: A: 2.5 mm; B: 500 μm; C: 50 μm; D: 2.5 mm; E: 50 μm.

By gross inspection, the volar MSTC donor wounds healed quickly, albeit with some erosion of the *stratum corneum* after 2 weeks which largely resolved to a normal appearance by week 3 post‐harvest. The animals were observed to mildly favor the post‐harvesting foot for the first 2–3 days after the procedure, although it is not clear whether that was due to pain at the donor sites, or just the general discomfort caused by the presence of dressing materials on that foot. No overt signs of pain or discomfort were observed beyond the third day, despite discontinuation of analgesic medications beyond that point. Through the end of the 8‐week study period, there were no clinical signs of scarring, or any other appreciable long‐term donor site morbidity, except for slight discoloration that appeared to be receding over time. At the histologic level, there were relatively minor but discernable changes at the donor sites. Collagen fibers in the donor sites were finer than in neighboring uninjured skin, and the neo‐epidermis had rete ridges that were similar in depth to uninjured skin, but generally reduced in width at the 8‐week timepoint (Figure 4).

### 3.4 Ectopic reconstitution of volar characteristics

**FIGURE 5:**
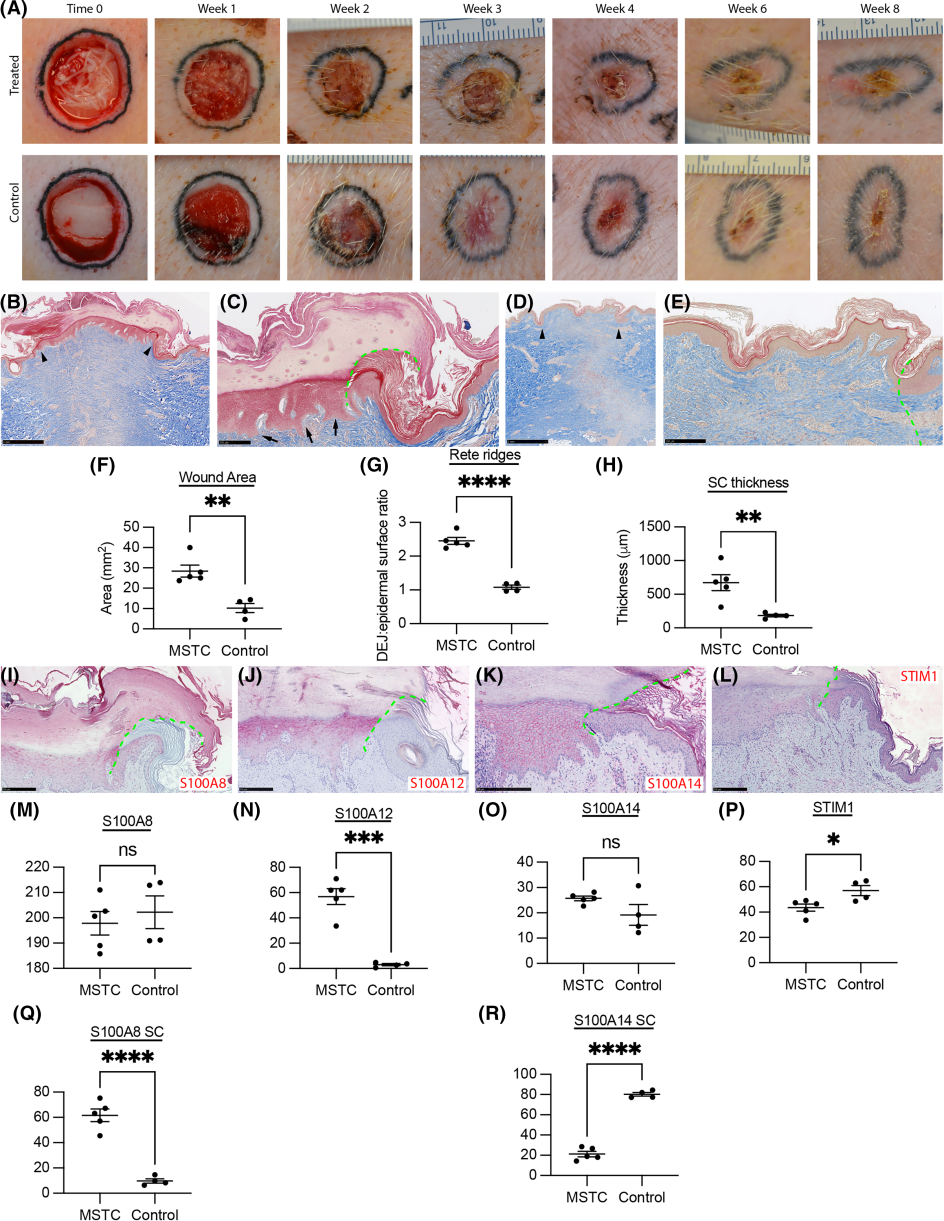
Volar characteristics recapitulated in ectopic graft sites. (A) Photographs of graft sites taken at various timepoints after injury and MSTC grafting, as denoted in the figure. Each photograph is shown at 2.5 mm across. The wound edges were tattooed with black ink to facilitate tracking of wound contraction. (B–E) Trichrome staining of graft sites at week 8. (B) Graft site treated with volar MSTCs, edges of graft site marked by arrowheads. (C) High‐power view of a volar MSTC‐treated graft site, focusing on the transition zone between the volar‐treated wound area (left of the dotted line) and the neighboring skin (right of the dotted line). Note substantially thickened epidermis, structurally compact stratum corneum, as well as pronounced rete ridges (arrows). (D) Control wound site histology, with edges of the injured area marked by arrowheads. (E) High‐power view of control wound, showing the transition zone between area of injury (left of the dotted line) and the neighboring skin (right of the dotted line). Note the epidermis and stratum corneum are similar in both thickness and structure between the injured area and surrounding skin, as well as the paucity of rete ridges in the area of injury. (F) Wound area measured at week 8, showing less contraction in the MSTC‐treated group compared to control. (G) The ratio between the lengths of the dermal‐epidermal junction (DEJ) and the top of the viable epidermis was taken to reflect the extent of rete ridges, which was about 2.5x greater in the volar MSTC‐treated wounds. (H) The stratum corneum was also significantly thicker in the volar MSTC‐treated wounds than in controls. (I–L) IHC staining of volar MSTC‐treated sites for various volar markers, as indicated in the images. Images are focused on the transition zone between the volar‐treated wound area (left of the dotted line) and the neighboring skin (right of the dotted line). (I) Strong S100A8 expression, most notably in the stratum corneum, in the volar MSTC‐treated wound area, but not in neighboring skin. (J) Expression of S100A12 only in the volar MSTC‐treated wound area, but not in neighboring skin. (K) The volar‐treated wound area shows strong pericellular S100A14 expression in the viable epidermis but weak expression in the stratum corneum, as opposed to the neighboring skin, where S100A14 expression is much stronger in the stratum corneum. (L) STIM1 expression is reduced in the volar MSTC‐treated wound area. (M–R) Quantification of IHC staining intensity for the different markers of interest. (M–P) show results from the viable epidermis. (Q and R) show results in the stratum corneum. In summary, the structural and molecular changes are consistent with characteristics of volar skin, and are highly specific to the volar MSTC‐treated areas, indicating that they are not a mere byproduct of the general wound healing response. *p < .05; **p < .01; ***p = .0001; ****p < .0001; ns, not significant. Scale bars: B: 1 mm; C: 250 μm; D: 1 mm; I–L: 250 μm.

Similar to our previous finding of enhanced healing in wounds treated with nonvolar MSTCs,^15^, ^18^ the treated wounds in this study showed faster wound re‐epithelialization and less contraction than untreated controls (Figure 5A,F). Histology at 8 weeks post‐treatment confirmed that trunk wounds treated with volar MSTCs reproduced the key structural characteristics of volar skin: the reconstituted epidermis was substantially thickened, the dermal‐epidermal junction featured pronounced rete ridges and the volar MSTC‐derived stratum corneum was also much thicker than its neighboring local trunk skin, with the highly compact structure that is a defining characteristic of volar skin (Figure 5B–E,G,H). The protein expression patterns of S100 markers and STIM1 in volar MSTC‐derived skin were also consistent with the distinctive presentation in volar skin, including increased protein levels of S100A8 and S100A14 in the stratum corneum, expression of S100A12 (normally only expressed in volar skin), and reduced STIM1 (Figure 5I–R). S100A8 and S100A14 in the viable epidermis were exceptions where expression was similar between MSTC‐treated and control sites. This may be because these volar markers are also induced by injury,^20^ and may remain elevated in the control wound sites during the normal wound healing process. In the border areas between volar MSTC‐derived skin and neighboring local (trunk) skin, the volar‐specific characteristics in structure and protein expression that were manifested in the volar MSTC‐treated areas all abruptly reverted to the typical nonvolar presentation in the adjacent local skin (Figure 5C,I–L). These volar characteristics were also not present in control wounds that were closed by secondary intention (not shown). These findings confirm that key volar characteristics can be stably conferred to ectopic recipient sites by treatment with volar MSTCs and that these characteristics are derived from the donor tissue, as opposed to being induced in nonvolar cells as part of the general response to injury/pathology (as some other volar skin features, most notably the expression of the human volar marker keratin 9, are known to do^10^, ^20^).

## 4 DISCUSSION

The goal of this study was to determine the possibility of using volar skin‐derived MSTCs to confer structural and molecular characteristics of volar skin ectopically onto other skin sites. Our results show that is indeed feasible to do so while avoiding the substantial and long‐lasting donor site morbidities that are the main drawback limiting (in most cases precluding) the use of autologous volar skin grafts. It has been previously reported that nonvolar skin grafts can be applied “randomly” without maintaining the skin's epidermal‐dermal orientation, as long as appropriate moisture levels are maintained in the recipient wound site, and that keratinocytes derived from the donor tissue are capable of spontaneously organizing and self‐assembling to re‐form a contiguous epidermis.^28^, ^29^ Hitherto it was not known if volar keratinocytes, which are physiologically distinct from their nonvolar counterparts, had the same capability. Our results from this study demonstrate that they do. This MSTC‐based approach—which could be completed as a bed‐side procedure in a few minutes in an outpatient setting—is likely to be both quicker and less costly than the autologous volar fibroblast culture/transplantation method (which requires access to sophisticated laboratory facilities and processes) that has been the focus of prevailing efforts to “volarize” stump skin. The two approaches could also have complementary applications—implantation of expanded autologous volar fibroblasts could prophylactically strengthen stump skin, while volar MSTCs may be particularly suited for repairing and augmenting areas of stump skin that have already experienced breakdown. In addition, due to the minimal morbidity involved, harvesting volar skin in the form of MSTCs could serve as a practical method of sourcing donor volar skin for the extraction of autologous volar cells, which in turn could have various applications including the aforementioned expansion and implantation of volar fibroblasts, and potentially for enhanced coverage of larger wounds by applying the cells in a spray form, similar to a previous approach to repair amputation stump wounds using nonvolar donor skin.^30^

Arguably the greatest drawback of conventional autologous skin grafting methods is donor site morbidity, which we have previously shown is greatly mitigated by the MSTC grafting approach in nonvolar porcine donor skin,^15^ and later verified independently in human subjects.^16^ However, it was not known whether volar MSTC donor wounds would follow a similar healing trajectory, especially in light of our recent finding that wound healing in volar skin is inherently delayed.^20^ In this study, we confirmed the lack of clinical signs of scarring or any other long‐term morbidity at volar MSTC donor sites, which is especially critical given the relatively small area and functional importance of volar skin regions. The formation of deep but thin rete ridges in the volar donor sites is an unexpected finding—it echoes the rapid restoration of rete ridges in volar wounds that we have previously observed^20^ and is a marked deviation from the effacement of rete ridges that is usually a hallmark of post‐injury scar formation. The apparent ability to spontaneously restore rete ridges in volar skin wounds may at least partially account for the common clinical observation that surgical incisions in plantar skin are generally able to heal with surprisingly little scarring.^31^ This aspect of volar wound healing is likely to be even more complicated (and more interesting to study) in humans because of the spatial heterogeneity in rete ridge architecture caused by the presence of friction ridges on the volar skin surface^32^ (thought to have evolved in primates and koalas to improve volar skin's gripping ability, which is especially beneficial in arboreal habitats^33^).

We showed that MSTC‐derived volar skin cells can integrate and survive in the treated wound site for up to 8 weeks. Future studies of increased durations will be needed to determine the feasibility of retaining these volar skin cells for longer times. It has been previously reported that volar‐derived cultured epidermal autografts can retain their volar‐specific characteristics for years after implantation in humans,^34^ but that was in an orthotopic setting where the autografts presumably could continue to benefit from paracrine factors and other local environmental cues that are native to volar skin. In heterotypic transplantation experiments, volar epidermis that was transplanted onto nonvolar dermis eventually took on characteristics corresponding to the anatomic origin of the underlying dermis.^9^ This suggests that the durability of the ectopic “volarization” effect conferred by our MSTC grafting approach is likely to be critically dependent on the continued presence and normal functioning of the volar‐derived fibroblasts. The same is likely to be true for the alternative volar fibroblast expansion/implantation approach. Thus the ability of volar fibroblasts (whether directly derived from MSTCs or expanded in culture) to persist and remain functional in ectopic skin sites will be an important metric to follow in clinical testing for either of these approaches. If repeated treatments are needed due to the limited durability of the ectopic volar characteristics, the ease of application, low production cost, and lack of donor site morbidity associated with the MSTC approach could be even more important beneficial factors.

In this study, we focused on the ability to confer key structural and molecular characteristics of volar skin to ectopic, nonvolar wound sites by the transplantation of autologous volar MSTCs. While the structure and composition of volar skin are thought to be critical in enabling the skin's weight‐bearing function,^7^ we did not directly evaluate such functional characteristics in this study. It may be difficult to specifically simulate the mechanical profile of weight bearing on stump skin in animal models, although previously developed assays for gauging skin's response to mechanical stress may be useful to gauge the functional properties of “volarized” skin in future studies.^35^ Alternatively, if the lack of long‐term donor site morbidities following MSTC harvests can be validated in human volar skin (as it has been in nonvolar skin^16^), then it may be ethically defensible to forgo further animal testing, but instead, test the approach directly in humans who are experiencing breakdown of stump skin.

In addition to “volarizing” and strengthening amputation stump skin, the volar MSTC harvesting/grafting approach also has potential applicability for repairing plantar wounds. Plantar ulcers are an enormous and still‐growing clinical and socioeconomic burden, with cost of care and mortality rates both surpassing those of many forms of cancer.^36^, ^37^, ^38^ Autologous split‐thickness skin grafting (STSG), where skin containing the epidermis and the upper portion of the dermis is harvested from the donor site and grafted onto the wound, is generally considered the “gold standard” for wound repair,^39^ and is highly effective for closing foot ulcers,^40^ but patients and physicians are often reluctant to apply this option, in large part due to concerns over donor site morbidities associated with skin harvesting, including acute and long‐term pain, risk of infection, discoloration, and scarring.^41^ The high recurrence risk for plantar ulcers undoubtedly makes the infliction of significant, and often permanent, donor site morbidities even less palatable. Indeed it has been estimated that of all the patients with foot ulcers who turned down STSG, nearly 70% did so due to aversion to donor site morbidities.^42^ In addition, volar skin wounds are best repaired with autologous donor tissue from volar skin, in order to avoid problems caused by structural and mechanical mismatches that occur when nonvolar skin is grafted onto volar wounds,^43^, ^44^ but the scarcity of volar skin available for harvest, and the fear of inducing additional nonhealing wounds at the donor site, generally precludes autologous volar skin grafts as an option for treating plantar ulcers. The healing of volar MSTC donor wounds has not been studied in patients with plantar ulcers, where various underlying pathologies hinder the healing process, but it has been reported that MSTC donor sites were able to heal normally even in the setting of pyoderma gangrenosum,^45^ which shows that the small size of MSTC donor wounds may make this a feasible option even in patients that are otherwise prone to impaired healing.

In summary, in this study, we have shown that MSTC grafting could be a practical approach to confer key structural and molecular characteristics of volar skin ectopically onto other skin areas, which could be applied to improve the load‐bearing capacity of amputation stump skin and enhance function, mobility, and quality‐of‐life for lower limb amputees. Future human testing is needed to provide definitive verification of this approach. The general MSTC harvesting and grafting technology has already been developed for clinical use,^16^, ^45^ and while some volar‐specific adaptations may be needed (e.g., using longer harvesting needles to account for the increased thickness of volar skin), the barriers against clinical utilization of the MSTC grafting approach for volar skin should be relatively low.

## AUTHOR CONTRIBUTIONS

R. R. Anderson, S. Cho, J. H. Meyerle, and J. Tam designed research. C. Fuchs, E. Wise, and J. Tam analyzed data. C. Fuchs, Y. Wang, W. A. Farinelli, and J. Tam performed research. R. R. Anderson, J. H. Meyerle, and J. Tam obtained funding. C. Fuchs and J. Tam wrote the paper. All authors reviewed the paper.

## DISCLOSURES

J.T., Y.W., W.F., and R.R.A. are co‐inventors on patents filed from the Massachusetts General Hospital related to the technology described in this manuscript, and through their institution the authors have received royalties and licensing revenue. These patents have been licensed to Medline Industries, LP, for which J.T. is a consultant. No commercial entity was involved in any aspect of this study, or the writing of this manuscript.

## DISCLAIMERS

This work was supported in part by the Military Medicine Technology Transformation Collaborative, Award Number HU0001‐17‐2‐0009. The Uniformed Services University of the Health Sciences (USU), 4301 Jones Bridge Rd., Bethesda, MD 20814–4799 is the awarding office and is administered through the Henry M. Jackson Foundation for the Advancement of Military Medicine, Inc. (HJF). The information or content and conclusions presented here do not necessarily represent the official position or policy of, nor should any official endorsement be inferred on the part of, USU, HJF, the Department of Defense, or the U.S. Government. RRA was partially supported by the Lancer Endowed Chair in Dermatology.

## Supporting information


Figure S1.

## Data Availability

The data that support the findings of this study are available in the Materials and Methods, Results, and Supplemental Material of this article.
