# Supplementary material for: Structural and molecular characteristics of weight‐bearing volar skin can be reconstituted by micro skin tissue column grafting
Source: FASEB J. 2024 Aug 6;38(15):e23873. doi: 10.1096/fj.202400866R (PMC11607627; doi:10.1096/fj.202400866R)
Supplement: Supplementary file 1 — Figure S1. [file FSB2-38-e23873-s001.docx]

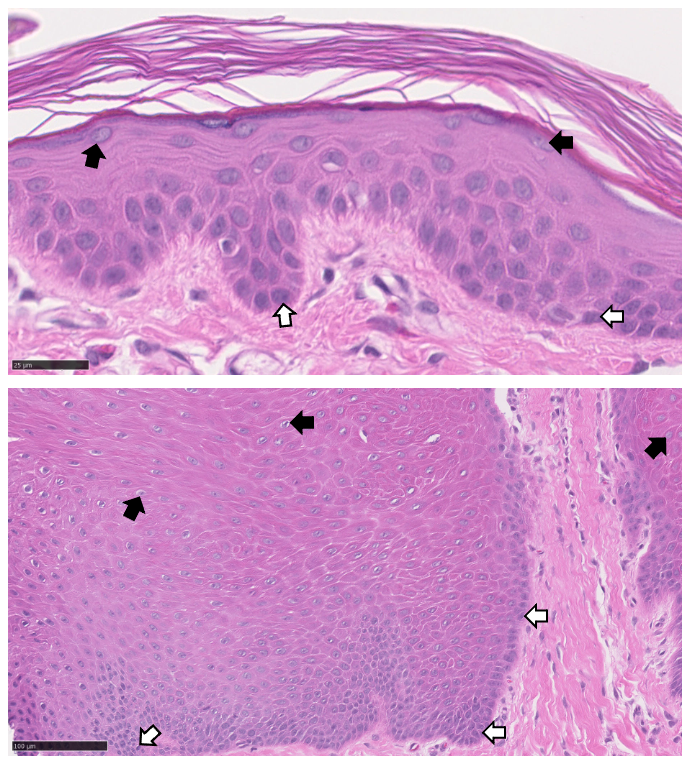


**Figure S1.** Variations in nuclear staining. H&E-stained samples from porcine trunk skin shown in the top image, porcine plantar skin in the bottom image. Note dense nuclear staining in basal keratinocytes (hollow arrows), which progressively fades away in the spinous and granular layers (solid arrows), as keratinocytes undergo terminal differentiation and ultimately become anuclear corneocytes. The partially disintegrated, lightly stained nuclei are more readily observable in plantar skin samples (bottom), due to the immense thickening of the suprabasal epidermal layers in plantar skin. Scale bars: top: 25 µm; bottom: 100 µm.


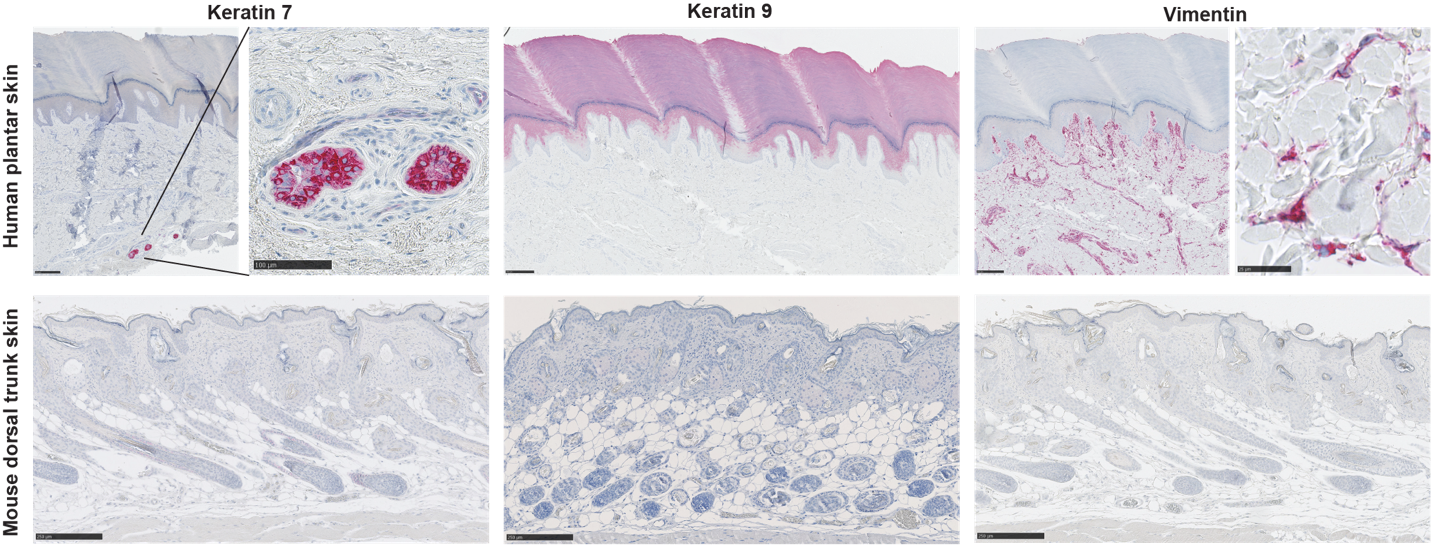


**Figure S2. Confirming human-specific reactivity of antibodies against keratin 7, keratin 9, and vimentin.** Human plantar skin tissue and murine dorsal trunk skin tissue samples were stained with antibodies against keratin 7, keratin 9, and vimentin, as denoted in the figure. As expected, in human plantar skin (top row) the keratin 7 antibody labeled eccrine sweat glands, the keratin 9 antibody stained the suprabasal epidermis, and the vimentin antibody stained dermal fibroblasts. None of the 3 antibodies showed any staining in the murine dorsal trunk skin samples. Based on these results, we conclude that the positively stained cells/structures shown in Figure 2 in the main text are derived from human MSTCs. Scale bars: 250 µm; high-power views: keratin 7: 100µm, vimentin: 25µm.


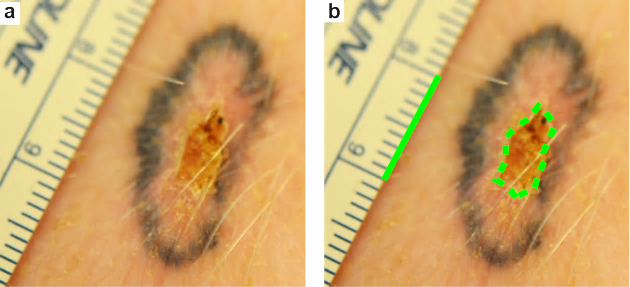


**Figure S3. Wound area measurement.** (a) Typical wound photo taken at week 8. (b) Ruler in each image used as scale (straight line) to measure wound area (dashed line) using Fiji. Results are shown in Figure 5f in the main text.


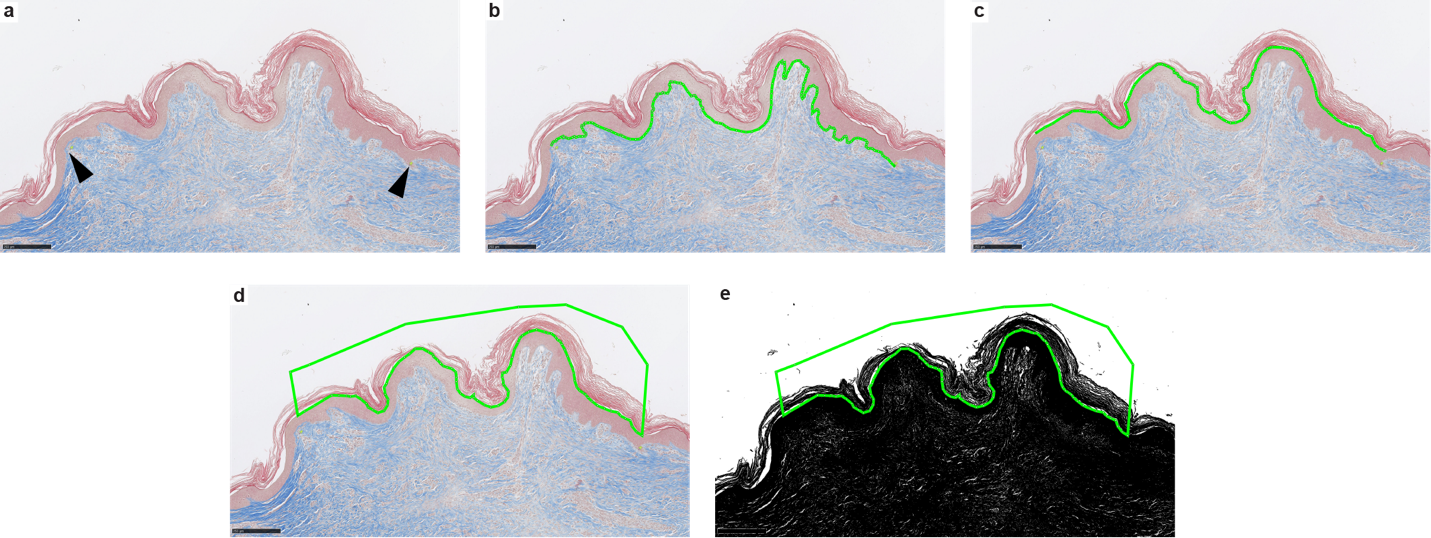


**Figure S4. Quantification of histologic features.** (a) Trichrome-stained image of a wound site biopsied at week 8, wound margins marked by arrowheads. (b) The dermal-epidermal junction (DEJ) marked in green (c) Top surface of the viable epidermis outline in green (the viable epidermis was used to measure the skin surface since the stratum corneum is often disrupted by the tissue sectioning process). The ratio between the DEJ (b) and epidermal surface (c) is taken to represent the extent of interdigitation between rete ridges and the underlying dermis. (d) The area containing the stratum corneum outlined in green. (e) Image of skin tissue is binarized by thresholding (black), the area occupied by the stratum corneum within the green outline is measured using Fiji. This area is divided by the epidermal surface length (c) to calculate the thickness of the epidermis. Image quantification results are shown in Figure 5 g-h in the main text. Scale bars: 250µm.

*
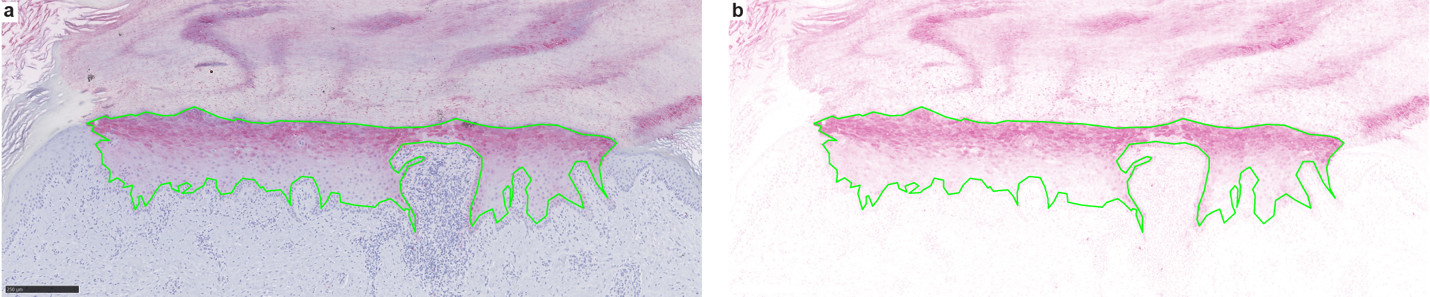
*

**Figure S5. Quantification of IHC staining.** (a) S100A12 staining in a porcine skin wound 8 weeks after treatment with volar MSTC is shown as an example. (b) A region of interest (ROI) is selected in the suprabasal viable epidermis of the repaired wound area (outlined in green). (b) The red-colored stain is separated from the other colors using Color Deconvolution 2 in Fiji. For STIM1 and S100A12, the average staining intensity within the ROI is then measured in Fiji. S100A8 and S100A14 had significant staining in the stratum corneum as well, therefore staining intensity was similarly quantified for the respective stratum corneum regions. For S100A14 in the viable suprabasal epidermis, the standard deviation in staining intensity was measured instead, due to the localization of S100A14 in the cell membranes (shown in the inset of Figure 3b). Image quantification results are shown in Figure 5 m-r in the main text. Scale bar: 250µm.
